# Characterisation of Leukocytes in a Human Skin Blister Model of Acute Inflammation and Resolution

**DOI:** 10.1371/journal.pone.0089375

**Published:** 2014-03-06

**Authors:** William Jenner, Madhur Motwani, Kristin Veighey, Justine Newson, Tatsiana Audzevich, Anna Nicolaou, Sharon Murphy, Raymond MacAllister, Derek W. Gilroy

**Affiliations:** 1 Centre for Clinical Pharmacology and Therapeutics, Division of Medicine, University College London, London, United Kingdom; 2 Manchester Pharmacy School, Faculty of Medical and Human Sciences, University of Manchester, Manchester, United Kingdom; McMaster University, Canada

## Abstract

There is an increasing need to understand the leukocytes and soluble mediators that drive acute inflammation and bring about its resolution in humans. We therefore carried out an extensive characterisation of the cantharidin skin blister model in healthy male volunteers. A novel fluorescence staining protocol was designed and implemented, which facilitated the identification of cell populations by flow cytometry. We observed that at the onset phase, 24 h after blister formation, the predominant cells were CD16^hi^/CD66b^+^ PMNs followed by HLA-DR^+^/CD14^+^ monocytes/macrophages, CD11c^+^ and CD141^+^ dendritic cells as well as Siglec-8^+^ eosinophils. CD3^+^ T cells, CD19^+^ B cells and CD56^+^ NK cells were also present, but in comparatively fewer numbers. During resolution, 72 h following blister induction, numbers of PMNs declined whilst the numbers of monocyte/macrophages remain unchanged, though they upregulated expression of CD16 and CD163. In contrast, the overall numbers of dendritic cells and Siglec-8^+^ eosinophils increased. Post hoc analysis of these data revealed that of the inflammatory cytokines measured, TNF-α but not IL-1β or IL-8 correlated with increased PMN numbers at the onset. Volunteers with the greatest PMN infiltration at onset displayed the fastest clearance rates for these cells at resolution. Collectively, these data provide insight into the cells that occupy acute resolving blister in humans, the soluble mediators that may control their influx as well as the phenotype of mononuclear phagocytes that predominate the resolution phase. Further use of this model will improve our understanding of the evolution and resolution of inflammation in humans, how defects in these over-lapping pathways may contribute to the variability in disease longevity/chronicity, and lends itself to the screen of putative anti-inflammatory or pro-resolution therapies.

## Introduction

Inflammation is characterised by the sequential release of mediators (including histamine, bradykinin and 5HT), resulting in the immediate influx of polymorphonuclear leukocytes (PMNs) followed by phagocytosing monocyte/macrophages, leading to leukocyte clearance and resolution [1]. Indeed, for the past 40 years research focused on identifying factors which initiate/perpetuate inflammation with the objective of developing drugs to alleviate diseases driven by on-going or dysregulated inflammation [2]. More recently, emphasis has shifted to the other end of the inflammatory spectrum, resolution, in order to understand how immune-mediated responses switch off. Advances in this area will help shed light on the aetiology of chronic inflammation and provide drug development opportunities based upon endogenous pro-resolution mediators/pathways [3]. However, elucidating the factors that drive inflammation, control its severity and longevity were, for the most part, characterised using rodent models of pleuritis, peritonitis or paw swelling [4], [5], [6], [7]. This included the response to innate (carrageenan) or specific (methylated bovine serum albumin) antigens.

In contrast, comparatively few human *in vivo* models of acute and resolving inflammation are available. Such models would allow us to better understand how the immune system is altered in people with chronic inflammatory diseases, and to determine the efficacy of novel immune-modifying agents. Performing such investigations requires existence of models that are representative of individual's innate inflammatory response, have low within-subject variability, and are non-invasive, such that they can be used appropriately in patients with exisiting inflammatory conditions. Of the human models currently in use for characterising and quantifying the inflammatory response, skin window techniques [8], and skin blisters induced by traumatic suction [9] or cantharidin [10] have proven useful in developing our understanding of the inflammatory phenotype. However, detailed analysis of trafficking cell populations that account for the onset and resolution of inflammation alongside traditional soluble mediators (cytokines and lipids) is lacking. These data would also confirm whether inherent mechanisms underlying the innate inflammatory response in humans are similar to those identified by rodent studies.

In the current study we therefore carried out detailed characterisation of leukocytes and soluble mediators occupying human cantharidin skin blisters at the onset of the inflammatory response and during its resolution.

## Materials and Methods

### Ethics Statement

This study was approved by the UCL ethics committee for human research (Ref: 2907/002). Written informed consent was obtained from all volunteers.

### Cantharidin blisters

The technique for inducing, aspirating, and processing the cantharidin skin blister and oedema has been previously described [11]. In short, blisters were elicited by applying 12.5 µl of 0.1% cantharidin (Cantharone, Dormer Laboratories) to the ventral aspect of the forearms of 20 non-smoking, healthy male volunteers aged 18–45 years. On day 1, two skin blisters were induced on one forearm with one blister aspirated on day 2 (24 hours) and the other on day 4 (72 hours). Peripheral blood samples were obtained following venepuncture at the antecubital fossa and leukocytes isolated following flash lysis to remove erythrocytes.

### Flow cytometry

Blister and circulating leukocytes were enumerated and then analysed for surface marker expression on a flow cytometer (LSR Fortessa, BD Biosciences). Due to a lack of published data on blister leukocyte differentiation using flow cytometry, a novel staining and subsequent gating strategy was designed to identify individual cell populations. Leukocytes were incubated with combinations of antibodies to *CD3* (APC, Clone: UCHT1, BD), *CD19* (PE-Cy 7, Clone: SJ25C1, BD), *CD56* (PerCP-Cy5.5, Clone: B159, BD), *HLA-DR* (V450, Clone: L243, BD), *CD14* (Alexa Fluor 700, Clone: M5E2, BD), *CD16* (FITC, Clone: 3G8, BD), *CD141* (PE, Clone: M80, Biolegend), *CD163* (PE, Clone: M130, BD), *CD11c* (PE-Cy7, Clone: B-ly6, BD), *Siglec-8* (PE, Clone:7C9, Biolegend) and *Annexin-V/7AAD* Apoptosis Detection Kit (BD) using respective isotype antibodies and fluorescence-minus-one (FMO) controls, and compensated for dual labelling. Separation of cell subtypes was performed using a cell sorter (FACS Aria, BD Biosciences) with subtypes undergoing histological staining using a modified Wright's method (Shandon Kwif-Diff Stain Kit, Thermo Scientific). Flow cytometry analysis was completed using FlowJo software (Tree Star Inc).

### Cytokine analysis

Cytokine expression profiles were measured using the MSD Bio-Plex human cytokine assay (Merck, Sharp & Dohme LTD). Our assay was customized to quantify the concentration of IL-1, IL-6, IL-8, IL-10, IFNγ, IL-12p70 and TNF-α within the blisters. All samples were run in duplicate.

### Extraction and analysis of lipid mediators

Lipid mediators in human plasma were analysed by liquid chromatography coupled to electrospray ionization tandem mass spectrometry (LC/ESI-MS/MS) based on protocols published previously [12], [13]. Briefly, samples were collected and stored immediately at −80°C. Plasma samples (500 µL) were defrosted on ice and adjusted to 15% (v/v) methanol: water (final volume 4 mL). Internal standards, PGB2-*d*4 (40 ng) and 12-HETE-*d*8 (40 ng) (Cayman Chemical Company, Ann Arbor, USA) were added and the pH of resulting solutions adjusted to 3.0 (1 M HCL). Acidified samples were immediately applied to preconditioned solid-phase cartridges (C18-E, Phenomenex, Macclesfield, UK) and lipid mediators eluted with methyl formate. LC/ESI-MS/MS analysis was performed on a HPLC pump (Waters Alliance 2695) coupled to an electrospray ionisation triple quadrupole mass spectrometer (Quattro Ultima, Waters, UK). Chromatographic separation was performed on a C18 Luna column (5 µm, 150×2.0 mm, 21 Phenomenex) for eicosanoids and a C18 Kinetex column (2.6 µm, 100×2.1 mm, Phenomenex) for hydroxy-fatty acids. Analytes were monitored on multiple reaction monitoring mode as reported [12], [13] with the following additions: 15-hydroxyeicosatrienoic acid (HETrE) *m/z* 321>221, 10-hydroxydocosahexaenoicacid (HDHA) *m/z* 343>153, 14-HDHA *m/z* 343>161, 13-HDHA *m/z* 343>193 and 17- HDHA *m/z* 343>201.

### Calculations and statistical analysis

Cell populations are expressed as the absolute number of cells (logarithmic scale, median ± interquartile range) and as the percentage of total cells (linear scale, mean ± standard deviation). Statistical analysis was performed using GraphPad Prism 4 (GraphPad Software). Between time-point differences in cells, cytokines, and lipids were assessed using paired *t* test for normally distributed data, or Wilcoxon matched pairs test for skewed data sets. Correlations between variables were calculated using Spearman's rank correlation. *p*<0.05 was considered statistically significant.

## Results

### Characterisation of peripheral blood leukocytes

**Figure 1:**
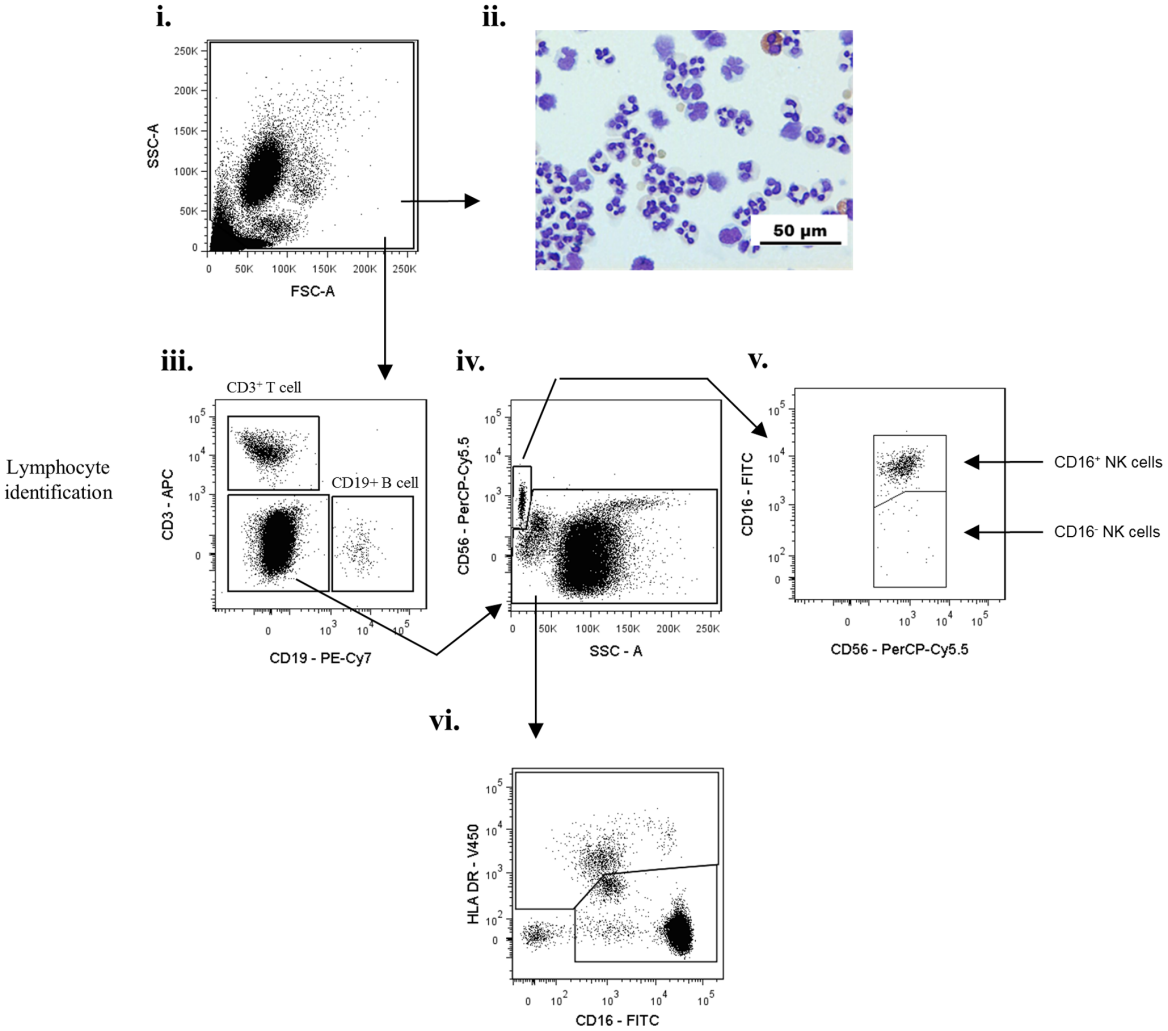
Characterisation of peripheral blood leukocytes from healthy volunteers- I. Representative dot plots for flow cytometric gating are shown for healthy male volunteers (n = 17). Blood was drawn from the forearm not bearing skin blisters. Erythrocytes were lysed and the remaining leukocytes incubated with antibodies and processed by flow cytometry. Gating strategies firstly identified CD3^+^ T cells, CD19^+^ B cells and CD56^+^ CD16^+/−^ NK cells. The remaining lymphocyte-deplete population was gated into HLA-DR^+^ and HLA-DR^−^ cells. Arrows indicate gating strategy.

**Figure 2:**
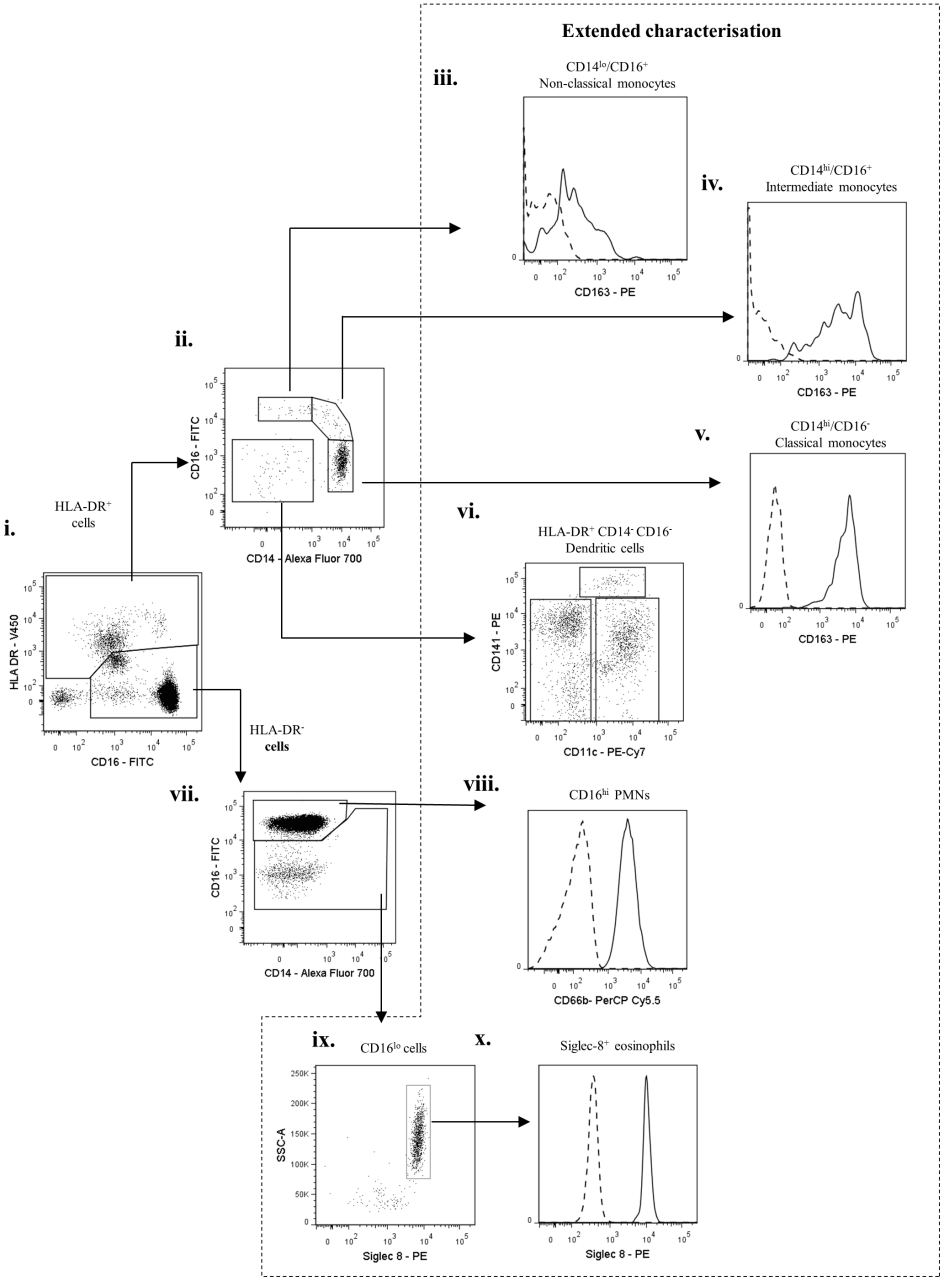
Characterisation of peripheral blood leukocytes from healthy volunteers- II. HLA-DR^+^ and HLA-DR^−^ cells identified in Figure 1 were further analysed. HLA-DR^+^ cells were characterised into CD14^hi^/CD16^−^ , CD14^hi^/CD16^+^, CD14^lo^/CD16^+^ monocytes and CD14^−^/CD16^−^ dendritic cells. HLA-DR^−^ cells comprised of typical PMNs (CD16^hi^, CD66^+^) and a CD16^lo^ population. On extended characterisation, HLA-DR^−^/CD16^lo^ cells were identified as Siglec-8^+^ eosinophils and the HLA-DR^+^/CD14^−^/CD16^−^ dendritic cells comprised of CD141^+^ and CD11c^+^ dendritic cell subpopulations. Arrows indicate gating strategy.

To develop an effective gating strategy for identification of inflammatory leukocyte sub-populations in skin blisters, we first validated the surface phenotype of known peripheral blood leukocyte populations by flow cytometry. After exclusion of cell debris and doublets (Figure 1 [i]), the remaining mixed cell population was firstly gated for CD3^+^ T cells and CD19^+^ B cells (Figure 1 [iii]). Resulting CD3^−^/CD19^−^ cells were then gated on CD56 (Figure 1 [iv]) and CD16 to identify CD16^+^ and CD16^−^ subpopulations of CD56^+^ NK cells (Figure 1 [v]). The remaining leukocytes were then differentiated on HLA-DR expression (Figure 1 [vi]). This allowed for separate classification of mononuclear and granulocytic populations, as has been previously described [14]. Analysis of HLA-DR^+^ cells (Figure 2 [i]) revealed the expected distribution of classical CD14^hi^/CD16^−^, intermediate CD14^hi^/CD16^+^, and non-classical CD14^lo^/CD16^+^ monocytes (Figure 2 [ii]), possessing varying degrees of the scavenger receptor marker, CD163 (Figure 1B [iii]–[v]). Within this HLA-DR^+^ population we also identified CD14^−^ CD16^−^ dendritic cells, which upon extended characterisation were identified as having mixed CD141 and CD11c expression (Figure 2 [vi]), as previously described [15].

The HLA-DR^−^ population (Figure 2 [i]) comprised of two sub-populations with varying degree of expression for CD16, labelled here as CD16^hi^ and CD16^lo^ (Figure 2 [vii]). CD16^hi^ population was identified as PMNs, confirmed by expression of CD66b (Figure 2 [viii]). On extended characterisation, the CD16^lo^ cells were identified as Siglec8^+^ eosinophils (Figure 2 [ix–x]).

### Characterisation of inflammatory cell infiltrates into skin blisters at 24 h - onset

**Figure 3:**
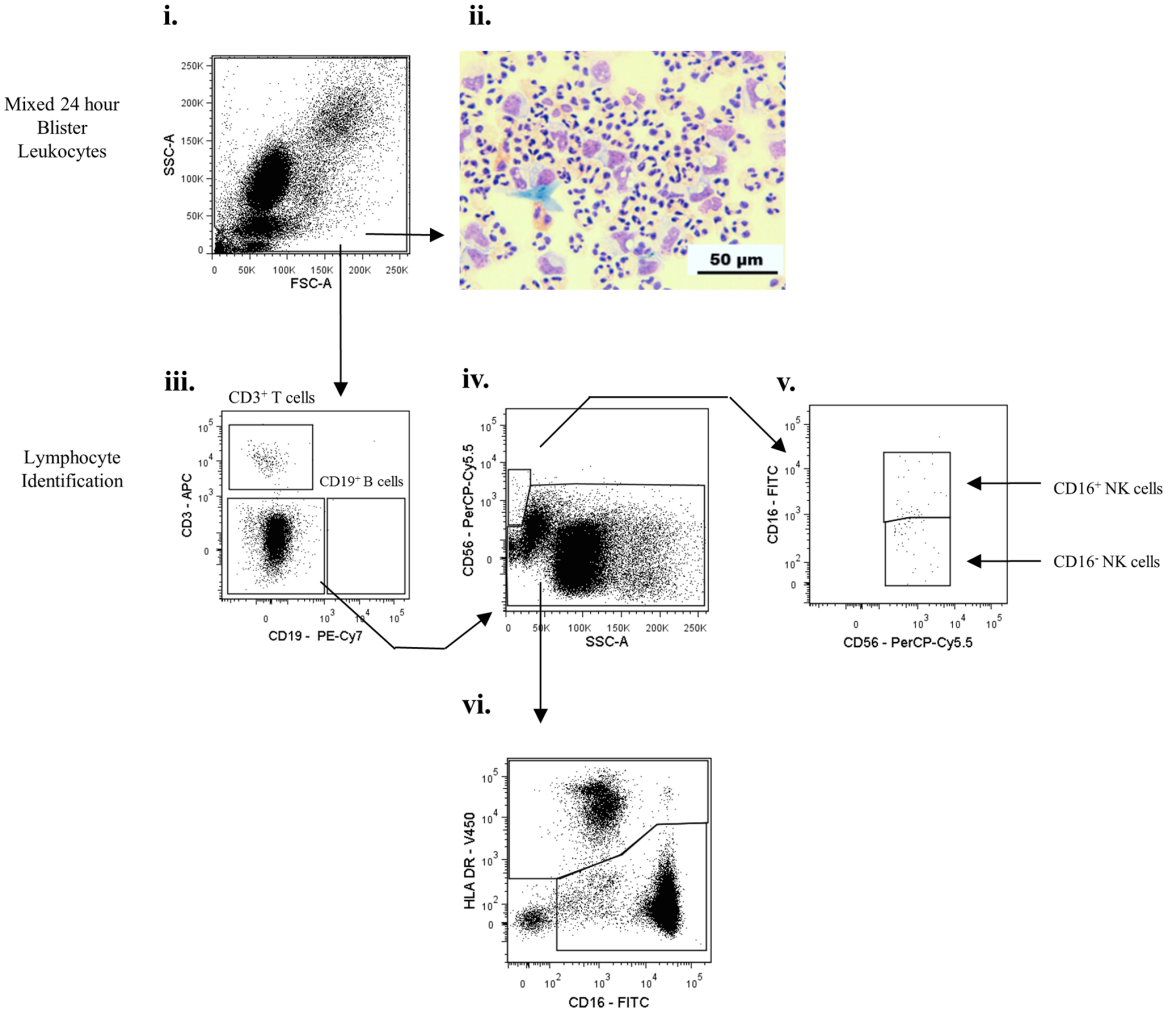
Characterisation of inflammatory cell infiltrates into skin blisters at 24(onset) – I. Representative dot plots for flow cytometric gating are shown for healthy male volunteers (n = 17). Blister contents were collected from one blister 24 h after application of cantharidin in 3% sodium citrate, with cells separated from oedema by centrifugation. Leukocytes were enumerated by haemocytometer and oedema volume recorded. Leukocytes were incubated with antibodies and processed by flow cytometry. Gating strategies firstly identified CD3^+^ T cells, CD19^+^ B cells and CD56^+^CD16^+/−^ NK cells. The remaining lymphocyte-deplete population was gated into HLA-DR^+^ and HLA-DR^−^ cells. Arrows indicate gating strategy.

**Figure 4:**
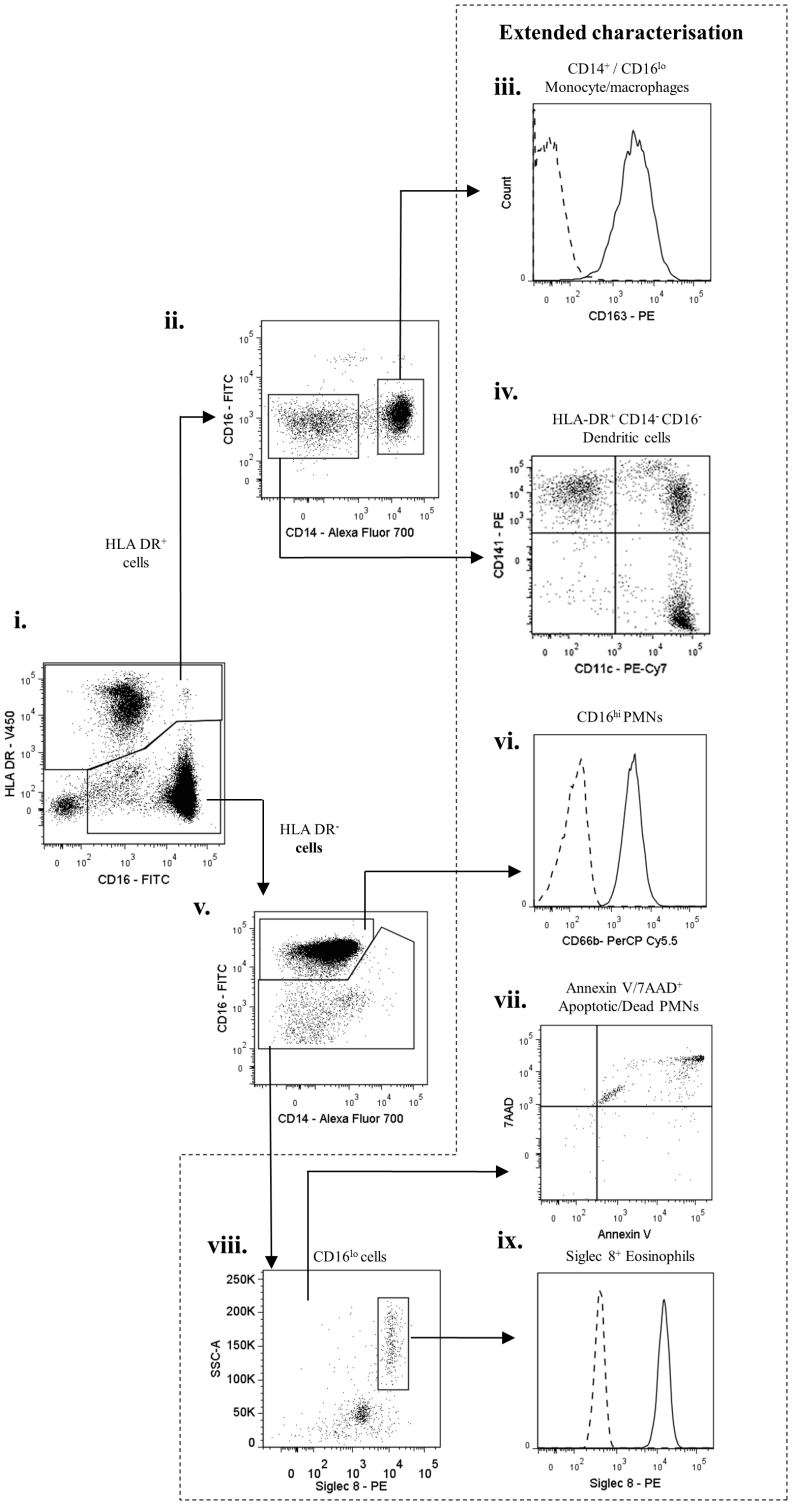
Characterisation of inflammatory cell infiltrates into skin blisters at 24(onset) – II. HLA-DR^+^ and HLA-DR^−^ cells identified in Figure 3 were further analysed. HLA-DR^+^ cells were characterised into CD14^+^/CD16^lo^ monocytes/macrophages and HLA-DR^+^/CD14^−^/CD16^−^ dendritic cells. HLA-DR^−^ cells comprised of typical PMNs (CD16^hi^, CD66^+^) and a CD16^lo^ population. On extended characterisation, HLA-DR^−^/CD16^lo^ were identified as a mixture of Siglec-8^+^ eosinophils and Annexin V/7AAD^+^ apoptotic/dead PMNs. The HLA-DR^+^/CD14^−^/CD16^−^ dendritic cells comprised of CD141^+^ and CD11c^+^ dendritic cell subpopulations. Arrows indicate gating strategy.

Skin blisters aspirated at 24 h following cantharidin application, showed a robust inflammatory cell infiltrate, as detected by histology (Figure 3 [ii]). By utilising the gating strategy developed for peripheral blood leukocytes, 24 h blister exudate revealed CD3^+^ T and CD19^+^ B lymphocytes (Figure 3 [iii]) and CD56^+^/CD16^+/−^ NK cells (Figure 3 [iv–v]). The remaining population was then probed for HLA-DR expression as for circulating leukocytes, and separated into the HLA-DR^+^ and HLA-DR^−^ cells (Figure 3 [vi]). The HLA-DR^+^ population (Figure 4 [ii]) comprised of CD14^+^/CD16^lo^ monocyte/macrophages expressing CD163 (Figure 4 [iii]), and a CD14^−^ CD16^−^ dendritic cell population, which upon extended characterisation, comprised of three dendritic cell subpopulations: CD11c^+^, CD141^+^, and CD11c^+^/CD141^+^ (Figure 4 [iv]).

The HLA-DR^−^ population comprised of CD16^hi^ and CD16^lo^ populations (Figure 4 [v]). CD16^hi^ population labelled positive for CD66b and was identified as PMNs (Figure 4 [vi]). The CD16^lo^ cells comprised two different populations with varying autofluorescence and side scatter. Flow-assisted cell sorting followed by modified Wright's staining on cytospin revealed these cells to be a mixture of eosinophils and apoptotic PMNs. The autofluorescent CD16^lo^SSC^hi^ population stained positive for Siglec-8 (Figure 4 [viii,ix]), whereas CD16^lo^SSC^lo^ population stained positive for apoptotic cell marker, Annexin-V/7AAD (Figure 4 [vii,viii]) and were characterised as apoptotic PMNs as previously reported [16]. Therefore, in a 24 h cantharidin-induced skin blister, PMNs are the predominant cell type along with monocytes/macrophages, dendritic cells and comparatively fewer lymphocytes and eosinophils.

### Characterisation of inflammatory cell infiltrates into skin blisters at 72 h –resolution

**Figure 5:**
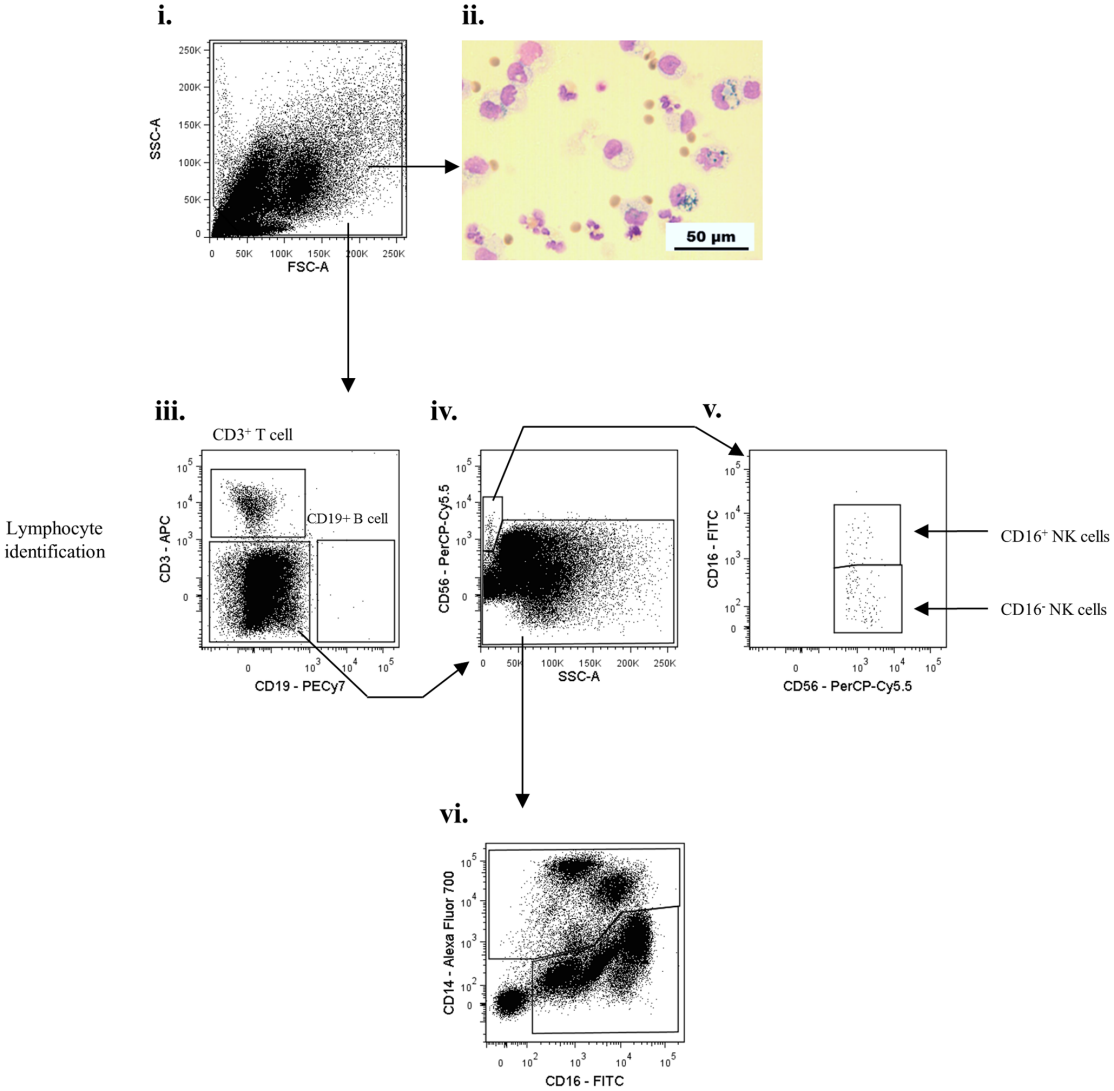
Characterisation of inflammatory cell infiltrates into skin blisters at 72(resolution) – I. Representative dot plots for flow cytometric gating are shown for healthy male volunteers (n = 17). Blister contents were collected from the remaining blister 72 h after application of cantharidin in 3% sodium citrate, with cells separated from oedema by centrifugation. Leukocytes were enumerated by haemocytometer and oedema volume recorded. Leukocytes were incubated with antibodies and processed by flow cytometry. Gating strategies firstly identified CD3^+^ T cells, CD19^+^ B cells and CD56^+^CD16^+/−^ NK cells. The remaining lymphocyte-deplete population was gated into HLA-DR^+^ and HLA-DR^−^ cells. Arrows indicate gating strategy.

**Figure 6:**
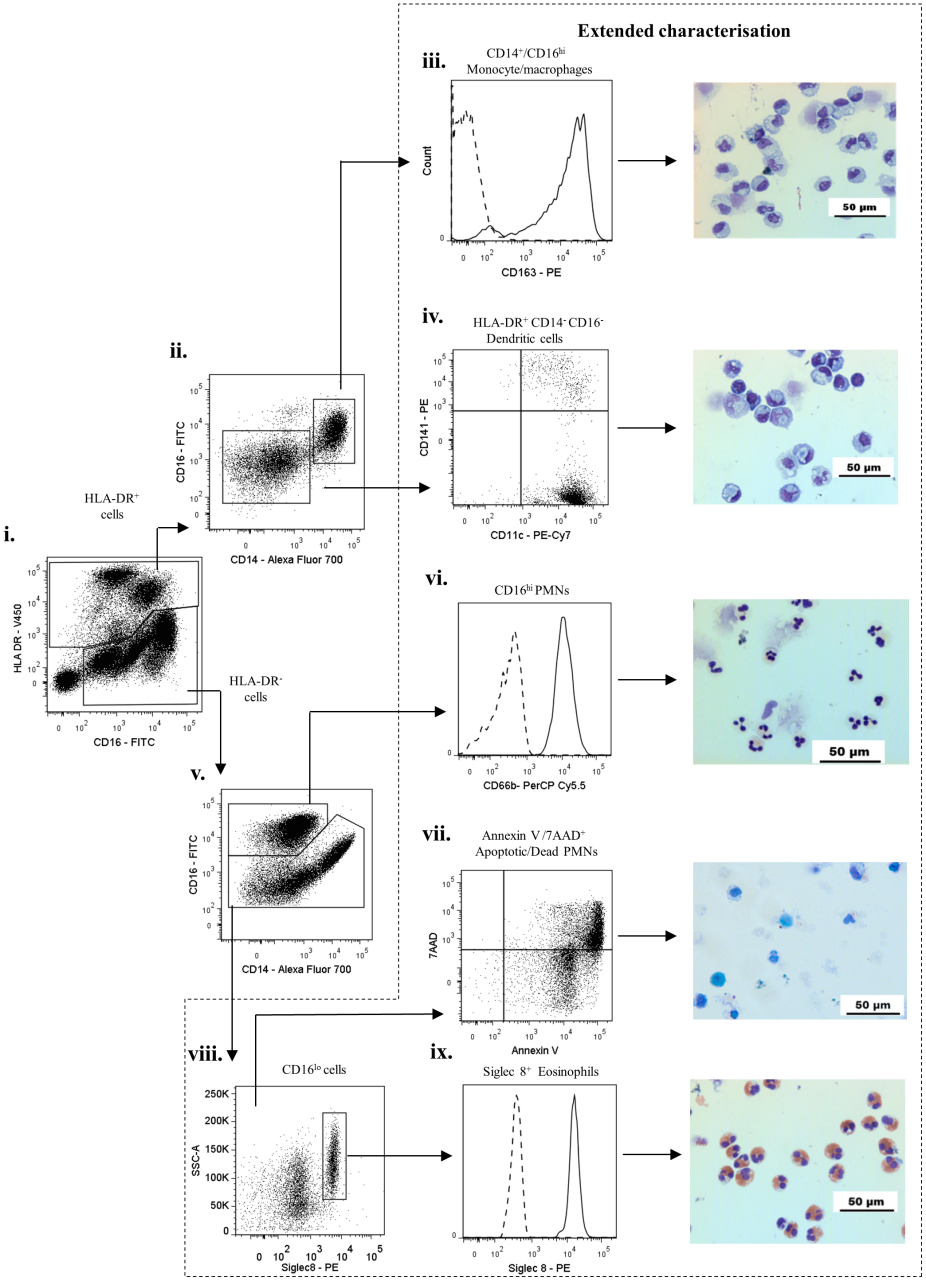
Characterisation of inflammatory cell infiltrates into skin blisters at 72(resolution) – II. HLA-DR^+^ and HLA-DR^−^ cells identified in Figure 5 were further analysed. HLA-DR^+^ cells were characterised into CD14^+^/CD16^hi^ monocytes/macrophages and CD14^−^ CD16^−^ dendritic cells. HLA-DR^−^ cells comprised of typical PMNs (CD16^hi^, CD66b^+^) and CD16^lo^ population. On extended characterisation, HLA-DR^−^/CD16^lo^ were identified as Siglec-8^+^ eosinophils and the HLA-DR^+^/CD14^−^/CD16^−^ dendritic cells comprised of CD141^+^ and CD11c^+^ dendritic cell subpopulations. Arrows indicate gating strategy.

**Figure 7:**
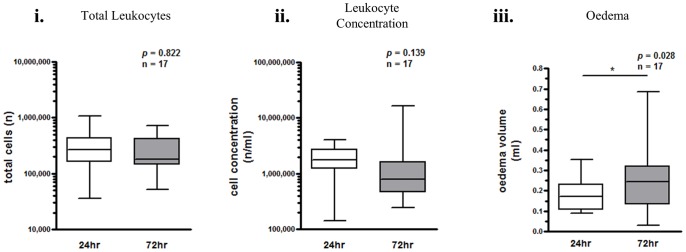
Total leukocyte and oedema profile in resolving skin blisters in humans. Blister content was collected in 3% sodium citrate 24 h and 72 h after application of cantharidin with cells separated from oedema by centrifugation. Red blood cells were lysed and the remaining leukocytes counted by haemocytometer while oedema volume recorded. Temporal differences from 24 to 72 h for total cells, leukocyte concentration and oedema volume are shown (n = 17).

**Figure 8:**
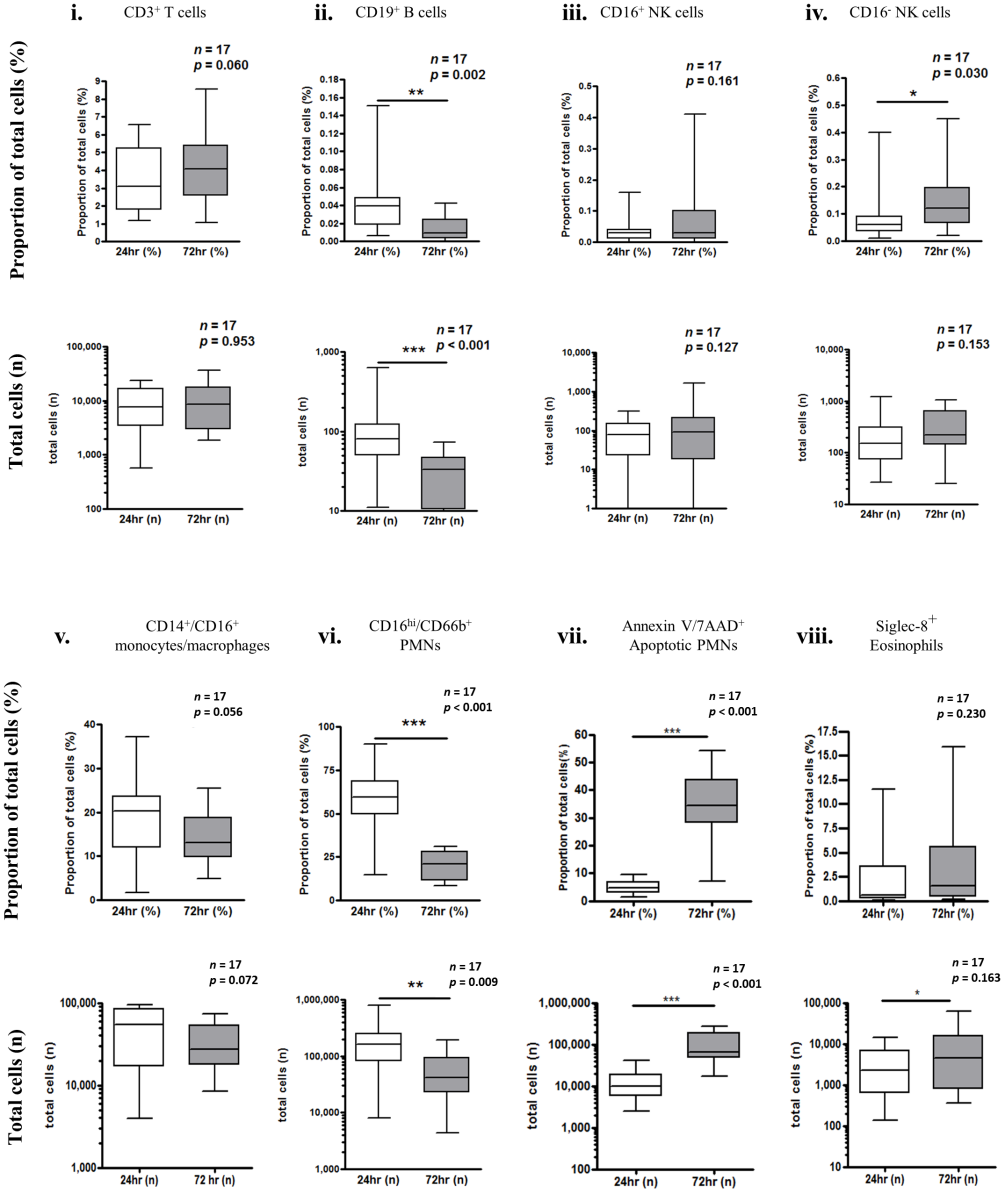
Leukocyte subtype profiles in resolving skin blisters in humans. Blister content was collected in 3% sodium citrate 24 h and 72 h after application of cantharidin with cells separated from oedema by centrifugation. Red blood cells were lysed and the remaining leukocytes counted by haemocytometer. Leukocytes were incubated with fluorescent antibodies, processed for flow cytometry, and gated according to Figures 2 and 3. Temporal changes in cell populations from 24 h to 72 h are shown (n = 17).

Flow cytometric characterisation of 72 h blister is outlined in Figure 5 and Figure 6. The gating strategy employed to 24 h blister was similarly applied at 72 h. In three individuals, one of the 24 h or 72 h blisters could not be aspirated, and as such the temporal changes in total leukocytes and oedema from 24 h to 72 h were quantified for 17 individuals (Figure 7). There was no overall difference in total leukocytes from 24–72 h (Figure 7 [i–ii]), but oedema volume increased significantly (Figure 7 [iii]). Individual leukocyte profiles show a decrease in CD19^+^ B cells (Figure 8 [ii]) and a modest increase in CD56^+^ NK cells (Figure 8 [iii–iv]). The absolute numbers of PMNs decreased significantly from 24 h to 72 h (Figure 8 [vi]). The number of monocytes/macrophages did not change significantly (Figure 8 [v]), but tended towards a reduction from 24–72 h. There was an increase in the number of apoptotic PMNs and eosinophils from 24 to 72 hours (Figure 8 [vii–viii]).

### Cytokines and lipid mediators

**Figure 9:**
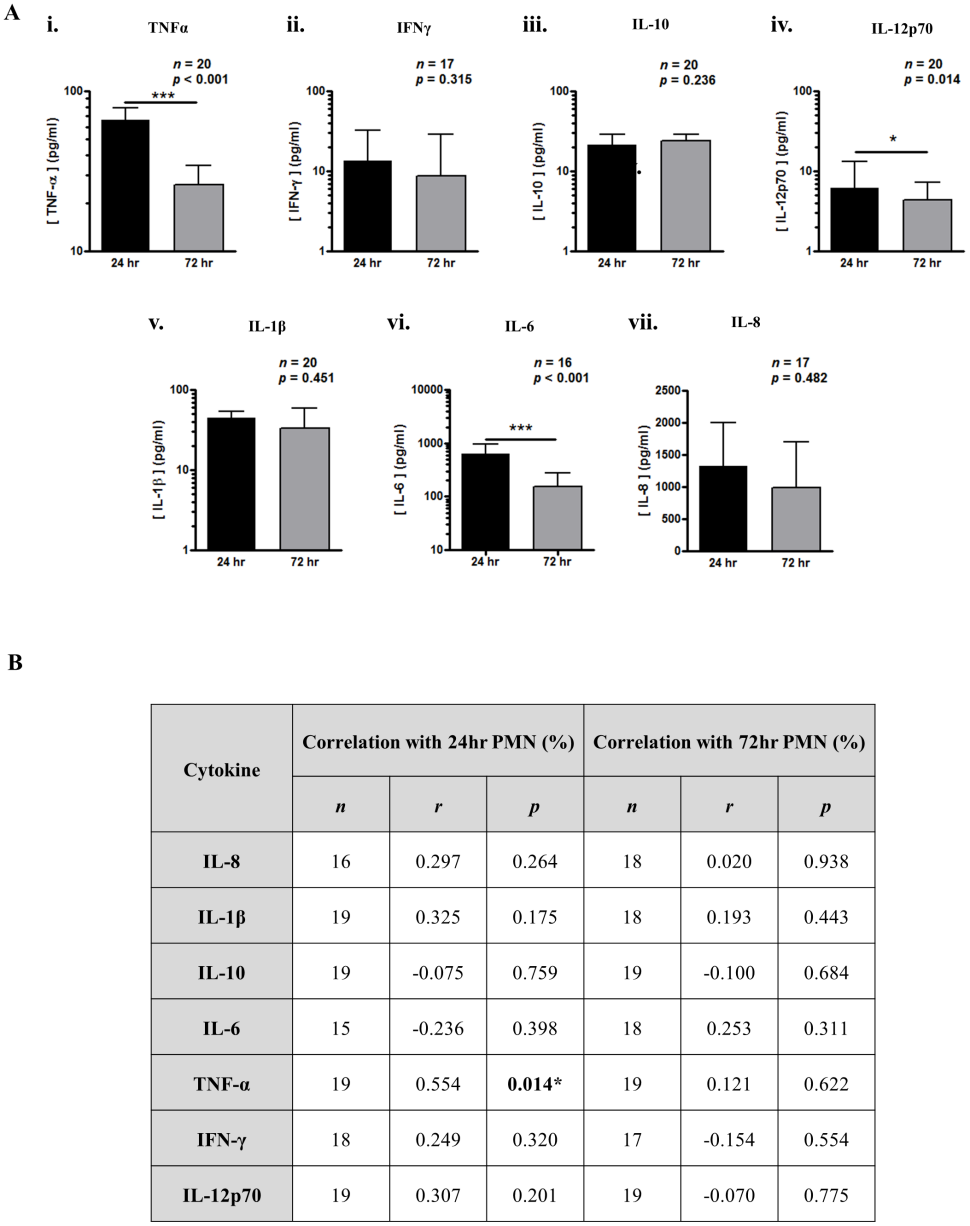
Cytokine profiles in resolving skin blisters in humans. Blister content was collected in 3% sodium citrate 24 h and 72 h after application of cantharidin with cells separated from oedema by centrifugation. Red blood cells were lysed and the remaining leukocytes counted by haemocytometer while oedema volume recorded and processed by assay for cytokine concentration (A). Correlations were made between cytokine concentration and PMN infiltration at 24 h and 72 h (B) (n = 17).

**Figure 10:**
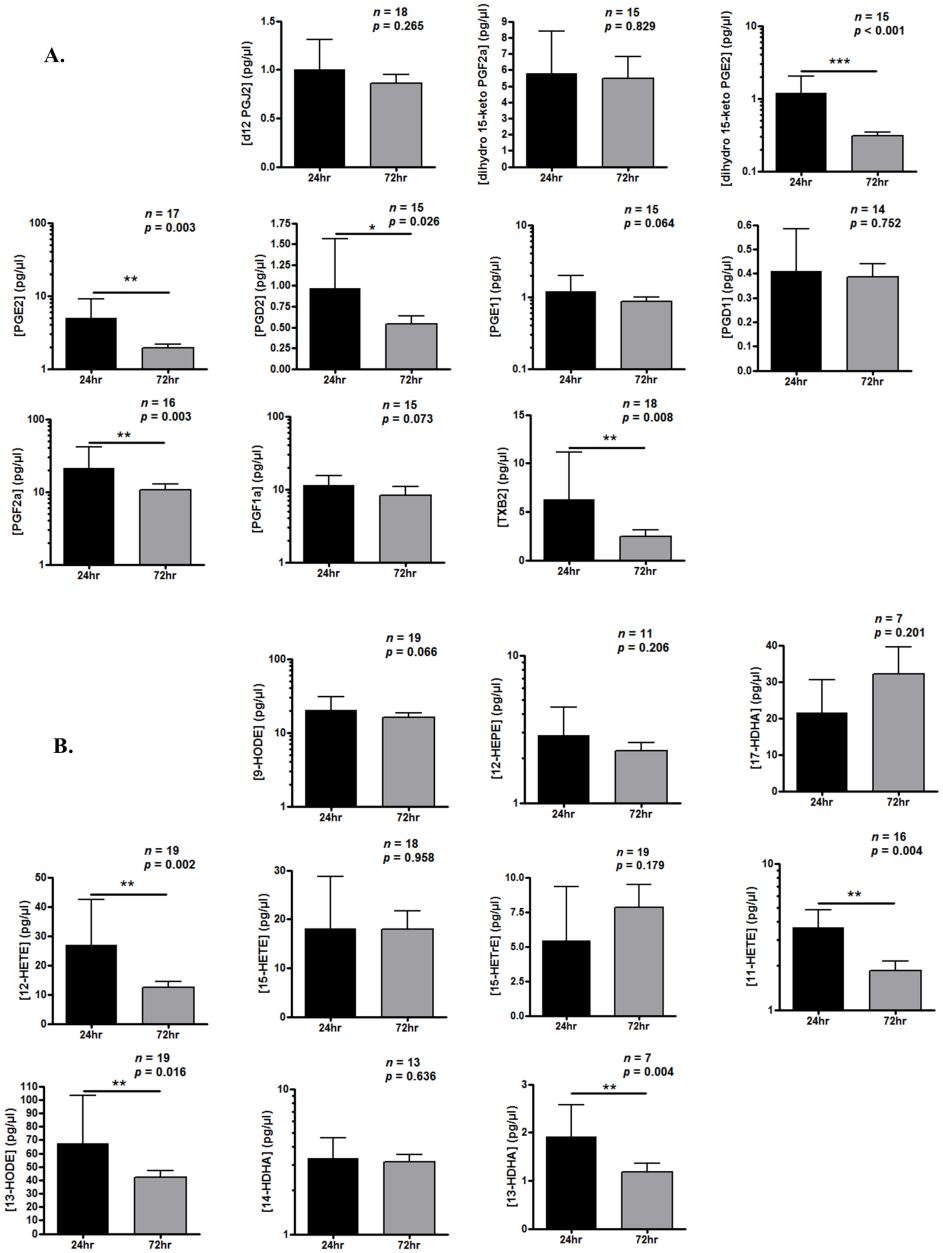
Lipid mediators in resolving skin blisters in humans. Blister content was collected in 3% sodium citrate 24 h and 72 h after application of cantharidin with cells separated from oedema by centrifugation. Red blood cells were lysed and the remaining leukocytes counted by haemocytometer while oedema volume recorded and analysed by liquid chromatography coupled to electrospray ionization tandem mass spectrometry for eicosanoid levels (n = 17).

There was an overall trend towards a reduction in typical pro-inflammatory cytokines from 24 h to 72 h, namely TNF-α, IL-6 and IL-12p70 (Figure 9A). Similar reductions were observed in lipid mediators, namely dihydro-15-keto PGE2, PGE2, PGF2a, TXB2 (Figure 10A), 12-HETE, 11-HETE, 13-HODE and 13-HDHA (Figure 10B). TNF-α levels positively correlated with PMN numbers at 24 h (Figure 9B). No such correlations were found at this early time between IL-8, IL-6 or IL-1β, and PMN trafficking or an inverse correlation with IL-10.

### Correlations in cells profiles from onset to resolution

**Figure 11:**
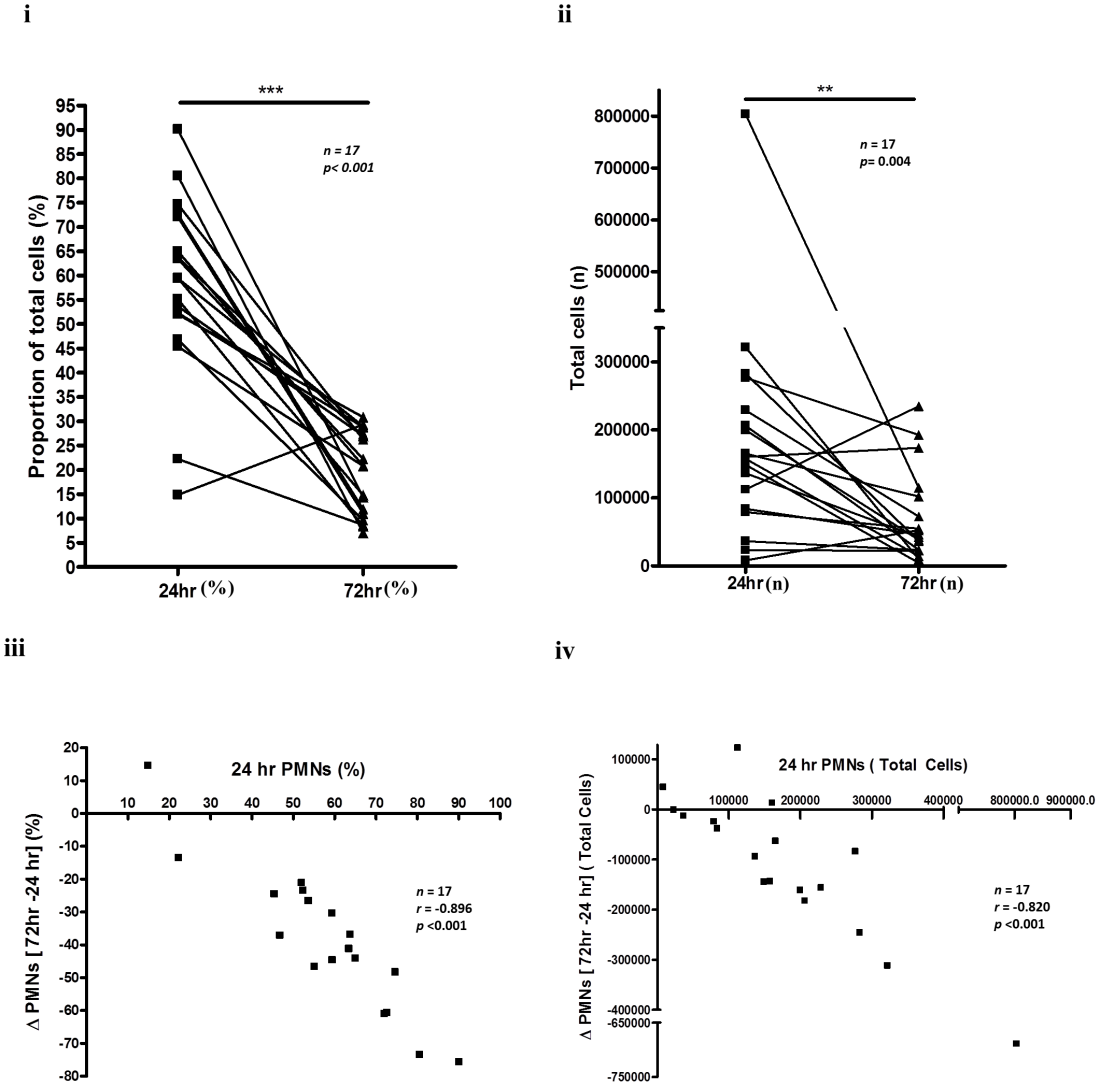
Correlations in cell profiles from onset to resolution. A wide range of PMN numbers infiltrate skin blisters early in the response (onset), and an immune response induces appropriate resolution. Correlation between change in PMN numbers (72 h minus 24 h) and total PMN numbers at onset are expressed as either total cells or as percentages.

At 24 h, absolute numbers and proportion of PMNs displayed a noticeable spread (Figure 11 [i, ii]). Subsequent comparison of 24 h PMNs with PMN clearance (calculated as 72 h PMNs - 24 h PMNs), revealed that volunteers with highest infiltration of PMNs at onset showed highest PMN clearance by resolution (Figure 11 [iii–iv]). Despite increases in other populations with putative pro-resolution properties including eosinophils [17], in this model of human acute inflammation we found no other significant correlations, inverse or otherwise, between PMN clearance and other cell types.

## Discussion

The current study presents an in depth flow cytometric characterisation of the cell trafficking within the cantharidin skin blister. The antibody panel and the gating strategy utilized here accurately identify the known populations of immune cells within the circulating blood [14]. This strategy was employed to identify the cells in cantharidin skin blister and was observed to be informative and accurate in conjunction with histology. The gating strategy serves as a template for future research to utilise the described model as a non-invasive tool to quantify and modify the human innate immune response *in vivo*.

One of the key observations from this model in terms of advancing our understanding of resolution in humans was that despite some individuals generating strikingly greater numbers of PMNs in their blisters at 24 h than others, all volunteers had approximately similar number of PMNs remaining at resolution. Thus, while some people elicit inflammatory responses of varying severity, infiltrating macrophages efficiently phagocytose these cells, generating a resolution phenotype that is appropriate for the given onset of inflammation.

The spread in PMN numbers at onset may result from the differential release in local TNF-α levels from stromal cells and/or tissue-resident macrophages. This is similar to work published previously using this model and its response to aspirin, whereby the more severe the inflammation, the more responsive it was to the anti-inflammatory effects of the NSAIDs [18], [19]. The rationale was that the greater the inflammation the corresponding high levels of pro-inflammatory/pro-resolution pathways would be modulated by aspirin. This is counter-intuitive as one would expect pharmacological agents to be proportionally more effective the less severe the inflammation. We argue that the efficacy of anti-inflammmatory agents is proportional to disease severity. Other key findings in this model were a lack of correlation between PMN influx and typical leukocyte chemoattractants such as IL-8 and the pro-inflammatory cytokine IL-1β. In addition, no inverse correlation was observed between IL-10 and inflammation severity at onset or key resolution indices such as decrement in PMNs from 24 to 72 h. It transpires that only TNF-α correlated directly with PMN infiltration supporting other findings reporting efficacy of anti-TNF drug in this model [19]. These observations suggest that of all the inflammatory cytokines and lipids, TNF-α has a more prominent role in controlling cell trafficking in acute inflammatory reaction in this skin injury model.

Furthermore study results identified an increase in overall numbers of dendritic cells, eosinophils and apoptotic PMNs during resolution. This offers insight into the potential importance of eosinophils in resolution of some inflammatory responses in humans. This theory supports recent murine studies showing that during resolution of acute zymosan-induced peritonitis, eosinophils congregate in the abdominal cavity and generate 15-lipoxygenase lipid mediators that are known to enhance the phagocytic ability of macrophages [20]. This must be borne in mind against the vast literature describing eosinophils as being pathogenic in allergic diseases [21].

Human peripheral blood monocytes are primarily classified into three main subtypes: classical CD14^hi^/CD16^−^, intermediate CD14^hi^/CD16^+^ and non-classical CD14^lo^/CD16^+^. Classical CD14^hi^/CD16^−^ monocytes constitute the majority of these cells types in peripheral blood and are considered to have anti-microbial roles and produce both pro-inflammatory and anti-inflammatory cytokines in response to LPS [22]. On the other hand, non-classical CD14^lo^/CD16^+^ monocytes are considered to resemble resident tissue macrophages with patrolling functions [23], [24]. They are the primary subtype that sense the presence of viruses and nucleic acids, and thereby produce pro-inflammatory cytokines such as TNF-α, IL-1β via TLR-8 and TLR-9 pathways [25]. Intermediate monocytes are specifically characterised as possessing highest levels of HLA-DR and are mainly involved in antigen presentation. Functional and gene array studies suggest that intermediate CD14^hi^/CD16^+^ monocytes share more in common with non-classical CD14^lo^/CD16^+^ monocytes than classical CD14^hi^/CD16^−^ monocytes [22]. CD16^+^ monocytes have been implicated in the pathogenesis of many diseases including sepsis [26], chronic liver disease [27], rheumatoid arthritis [28], atherosclerosis [29], tuberculosis [30], [31] , and HIV [32], [33]. In the current study, all three subtypes were identified in circulating leukocytes, however in skin blisters at 24 h we noted that HLA-DR^+^/CD14^+^ monocyte/macrophage population predominated and had low expression of CD16. Surprisingly, as inflammation resolves, numbers of HLA-DR^+^ CD14^+^ monocyte/macrophages remained stable or were slightly reduced, but gained CD16 expression. It is uncertain whether these are resident cells, a newly influxed non-classical/intermediate monocyte population or a classical monocyte-derived macrophage population that gain CD16 expression. Nonetheless, presence of these cells in a large quantity appear to be associated with the clearance of PMNs, and can be assumed to be a type of pro-resolution macrophages in humans, also reported recently [34].

A number of challenges were encountered during the design of cell differentiation by flow cytometry. During eosinophil identification, we observed that choice of fluorochrome can cause a gating error due to auto-fluorescence. For instance, eosinophils have alkaline cytoplasm and do not express CD16. In this study we used the acidic fluorochrome, fluorescein isothiocynate (FITC) tagged to anti-CD16 antibody to identify PMNs/monocytes/NK cells and observed that the alkaline cytoplasm of eosinophils non-specifically bound to FITC giving the erroneous impression that eosinophils express CD16, as others have previously reported [35]. Thus, care must be taken when trying to identify eosinophils within mixed cell populations using FITC-labelled antibodies and we urge the use of Siglec-8 antibodies to identify bono fide eosinophils, and to avoid such errors.

In conclusion, this study has thoroughly characterised the cells of the innate immune system that occupy a self-resolving inflammatory response in healthy individuals. It identified a predominance of PMNs at onset and an increase in eosinophils and dendritic cells during resolution. The advantage of this model is that it is non-invasive, inexpensive, has low within-subject variability, and provides immediate access to cells in a manner that minimises alteration in phenotype arising from *ex vivo* processing. The important caveats to this model are that it is not driven by a defined antigen and must be regarded as a model of tissue injury as a consequence of cantharidin-driven acantholysis.
